# The Effect of Sterilization on the Accuracy and Fit of 3D-Printed Surgical Guides

**DOI:** 10.3390/ma16155305

**Published:** 2023-07-28

**Authors:** Christine Yazigi, M. Sad Chaar, Reinhard Busch, Matthias Kern

**Affiliations:** Department of Prosthodontics, Propaedeutics and Dental Materials, School of Dentistry, Christian-Albrechts University, 24105 Kiel, Germany; schaar@proth.uni-kiel.de (M.S.C.); rbusch@proth.uni-kiel.de (R.B.); mkern@proth.uni-kiel.de (M.K.)

**Keywords:** implants, guided implant placement, surgical guides, additive manufacturing, 3D-printing materials, sterilization, accuracy, classification of medical products

## Abstract

This study was conducted to evaluate the accuracy of 3D-printed surgical guides before and after sterilization in a steam sterilizer. A test-model incorporating three implant replicas was customized. A total of forty guides were printed from five printable resins. A group made from a self-curing composite served as control group. The guides were checked for fit. Vertical discrepancies between the model and guides were measured at standardized points at a load of 500 g (P1). The guides were connected to implant replicas and scanned, and their angles were digitally measured. The specimens were sterilized in a steam sterilizer at 121 °C for 20 min at 2 bar pressure. Vertical discrepancies (P2) and angulations were remeasured. Additionally, the specimens were repositioned with an increased load, and measurements were repeated (P3). All specimens were repositionable after sterilization. The smallest variation in discrepancy at a 500 g load was 428 µm, whereas the greatest was 1487 µm. Under an increased force, the smallest change was 94 µm, while the greatest was 260 µm. The level of significance α = 0.05 (95% confidence interval) was set for all tests. The variation in the measured angles was not statistically significant (Kruskal–Wallis’s test, *p* > 0.05). The accuracy was affected by the material and sterilization, but it was clinically acceptable when an increased load was applied during repositioning.

## 1. Introduction

Implant-supported restorations are becoming increasingly popular in contemporary dentistry [1]. They represent an alternative treatment modality as compared to conventional restorations and prostheses in cases of missing teeth. High clinical success rates as well as survival rates are documented for implant-supported restorations [2,3,4,5,6].

Placing a successful implant requires applying precise laboratory and clinical steps [3]. The clinical procedure starts with the selection of patients according to each patient’s individual risk factors, following the right indications in addition to an ideal 3D planning and positioning of the implant in order to conduct a successful treatment [3,7,8]. 

The correct positioning of implants takes into consideration anatomical, biological and esthetic considerations to minimize possible biological and technical complications [2,9,10]. Accurate implant positioning can be achieved by guided implant insertion using surgical guides that transfer the virtual implant plan to the surgical site through the integration of cone beam computed tomography (CBCT) and 3D planning software [2]. 

Surgical guides can optimize implant placement and minimize operational errors and possible complications provided that they are accurate, stable and rigid [1]. A randomized clinical study showed that a maximum of a 2 mm apical deviation from the ideal implant position can be achieved by means of fully guided surgery and 3 mm for pilot-drill-guided surgery in comparison to a maximum deviation of 5 mm for free-handed surgery [2]. Moreover, guided implant surgery can be implemented in order to achieve a minimally invasive implantation procedure such as flapless implantation, minimizing the patients’ discomfort, surgical time and post-operative pain [11,12,13]. 

Surgical guides can be categorized according to the following: fabrication material, design, production technique, support type, implemented protocol and guidance system [2,12,14,15]. Three-dimensional printing or additive manufacturing is a modern revolutionary technology that has been incorporated in the field of dentistry with promising perspectives. This technology is being used for the manufacturing of modern surgical guides that enjoy high levels of precision [16]. However, there is no defined classification for surgical guides nor for their fabrication materials as a medicinal product nor a validated defined procedure for a recommended infection-control protocol. The results of a questionnaire performed in Germany in 2008 showed that out of 100 participants using surgical guides for implantation procedures, 99 of them used disinfection rather than sterilization as the infection-control method. The most-used disinfection solutions were CHX solution (30%), alcohol (23%) and Octenidin (7%) [17]. 

Although a surgical guide is individually manufactured in a dental laboratory for a single use for a patient, it nevertheless might become contaminated through many possible sources during the manufacturing process in the dental lab. The most common microorganisms that can be transmitted in a dental laboratory are *Pseudomonas aeruginosa*, *Acinetobacter baumannii*, *Enterococcus faecalis*, *Enterococcus faecium*, *Staphylococcus aureus*, *Enterobacter cloacae*, *Escherichia coli* and *Candida albicans* [17]. A surgical guide is then used for the surgical intervention, which means it comes into contact with the open wound area and exposed bone in addition to the bloodstream during this process. Accordingly, to avoid the risk of infection and post-operative complications and to increase the success of the surgery and that of the implant, surgical guides should be classified as class IIb medicinal products that should be sterile when used in order to protect the patient, the practitioner and any third party from any contamination or risk of infection [17,18,19]. However, some of the most-used printing materials for surgical guides are not even categorized as sterilizable [16]. 

Many sterilization methods have been used in dental practice such as dry heat sterilization, steam sterilization and radiation sterilization, such as gamma and X-ray irradiation [20,21,22]. Dry heat sterilization is non-toxic and environmentally friendly, but it needs high heat for long periods [23]. X-ray sterilization or bremsstrahlung is chemical-free and radioactivity-free and can be used efficiently for large loads with low-density packages. However, studies have shown a negative impact of this method on the properties of polymer materials [24]. Gamma radiation sterilization has the advantage of high penetrability, low reactivity, no toxic residues and time and cost effectiveness. Its disadvantages include the possible effects on the materials’ properties such as discoloration, stiffening, softening, embrittlement and the need for a strict validation of the sterilization dose that has to be determined specifically [25,26,27]. Steam sterilization is still the most commonly used sterilization method in dental practice due to its low cost, convenience and proven sterilization effect [21,22,28]. 

To the best knowledge of the authors, the possible effects of the sterilization process on the dimensional stability of 3D-printed surgical guides has been scarcely addressed [29]. The aim of this study was therefore to measure and compare the accuracy of 3D-printed surgical guides made from five different 3D-printing materials before and after steam sterilization in an autoclave at 121 °C and at a pressure of 2 bar for 20 min. The first null hypothesis was that no dimensional changes would occur after sterilization. The second null hypothesis was that the choice of material would have no significant effect on the dimensional changes caused by sterilization, if applicable.

## 2. Materials and Methods

**Figure 1:**
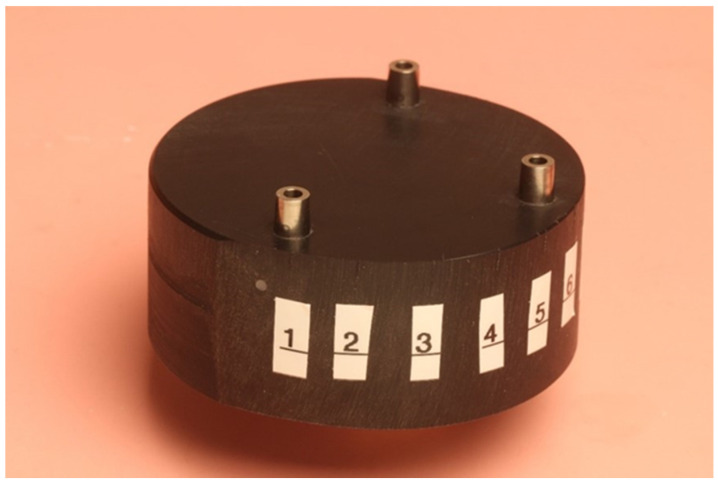
Test model.

A test model of polyoxymethylen (Delrin) was fabricated. Three implant replicas (Astra Tech Implant System; Dentsply, Mannheim, Germany) were incorporated in the test model, one in the front and one on each side. Eleven points were marked and numbered on the test model for future measurements. The test model is illustrated in Figure 1. 

**Figure 2:**
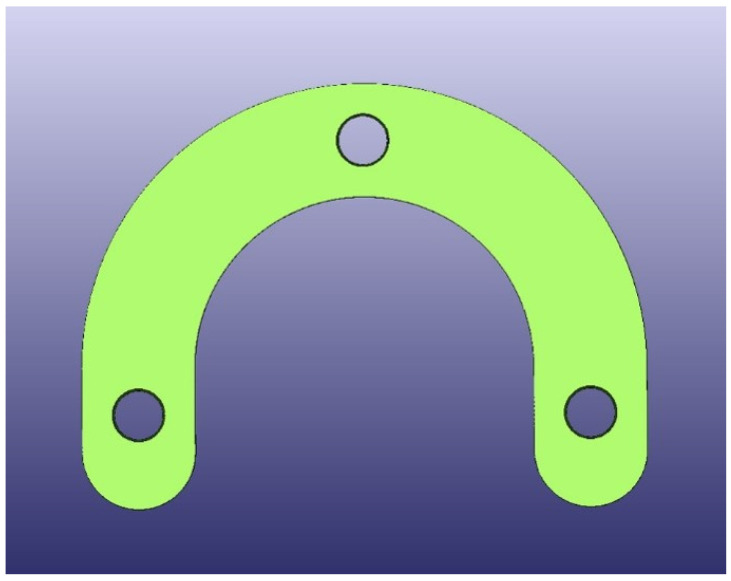
STL design of guides.

**Figure 3:**
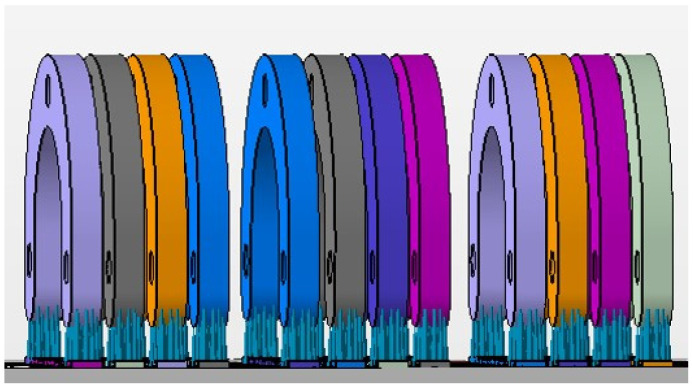
The vertical digital arrangement before printing.

**Figure 4:**
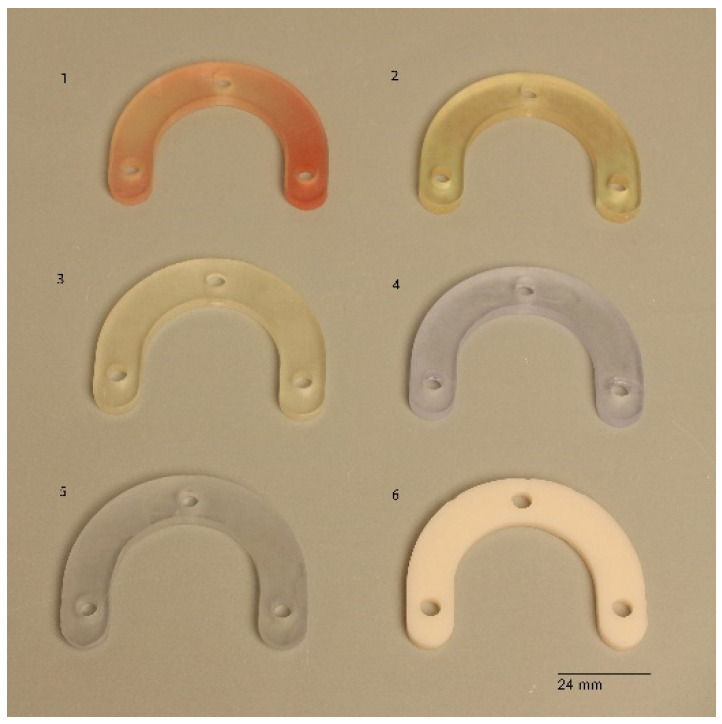
Specimens of the 6 test materials. 1: Gr. S, 2: Gr. N, 3: Gr. V, 4: Gr. L, 5: Gr. O, 6: Gr. C.

The test model was digitally scanned using a 3D scanner (D900 3D scanner; 3Shape, Copenhagen, Denmark: 4 cameras at 5.0-megapixel resolution, blue LED light technology and a documented scan accuracy of 7 microns for crown and bridge and of 8 microns for implant bars). A surgical guide with a thickness of 5 mm and a convergence of 2.5° for the replicas’ space holes was designed using the CAD/CAM software (3Shape DentalDesigner Premium 2013, 3Shape) and saved as an STL file (Figure 2). The design was exported as an STL file and was 3D-printed using 5 different 3D-printing materials (S: Sheraprint-SG; Shera Werkstoff-Technologie GmbH & Co. KG, Lemförde, Germany; N: NextDent SG; 3D Systems GmbH, Moerfelden-Walldorf, Germany; V: V-Print SG; Voco, Cuxhaven, Germany; O: Optiprint guide 385; Dentona AG, Dortmund, Germany; and L: LuxaPrint Ortho; DMG Chemisch-Pharmazeutische Fabrik GmbH, Hamburg, Germany). Eight specimens were printed for each group of material. The vertical digital arrangement of the surgical guides before printing can be seen in Figure 3. The 3D-printing was conducted according to the recommendations of the manufacturer and with the recommended 3D printer through digital light processing (DLP). An additional group, which was used as a control group for comparison purposes, was made from self-curing composite material (C: Luxatemp Fluorescence; DMG Chemisch-Pharmazeutische Fabrik GmbH). For this purpose, a duplicated silicone form was produced. The groups with their respective codes as well as the manufacturer and 3D printer are shown in Table 1. Specimens of the six tested materials are illustrated in Figure 4.

**Figure 5:**
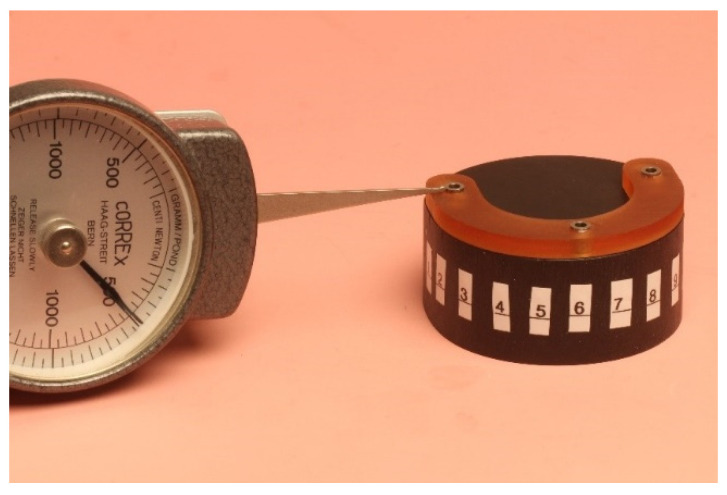
Positioning of the guide onto the test model with a load of 500 g.

**Figure 6:**
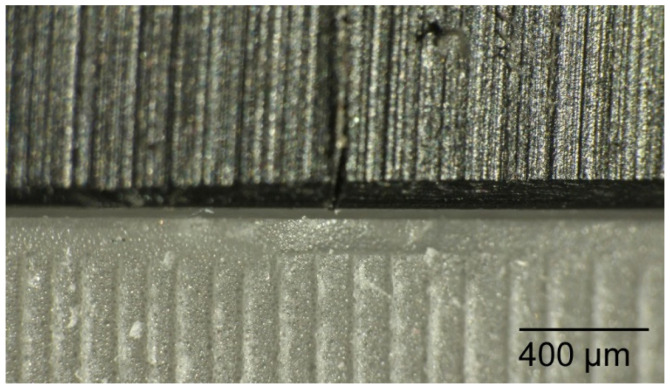
The resulting vertical discrepancy to measure between the guide and the model checked under an optical microscope.

Each surgical guide was positioned onto a test model to prove that it fitted passively and accurately on the test model. For the measurements of the initial space between the guides and the test model, each surgical guide was fitted again onto the test model with a load of 500 g using the drag pointer of a measuring instrument (Correx; Haag-Streit, Bern, Switzerland). The load was applied at three specified points in front of the implants that were standardized for all guides (Figure 5). The resulting vertical discrepancy between the guide and the model at a load of 500 g was checked under an optical microscope with a 16× magnification (Wild M420; Heerbrugg, Switzerland) for all 11 standardized marked points, and a corresponding photo of the section was made (Figure 6). The measurements were conducted with photo editing software (Adobe Photoshop CC 19.1.6 release; Adobe Inc., San Jose, CA, USA). Each section was measured 10 times, and the resulting mean value was set as the vertical discrepancy for each mark between the guide and the model before sterilization at a load of 500 g (P1).

**Figure 7:**
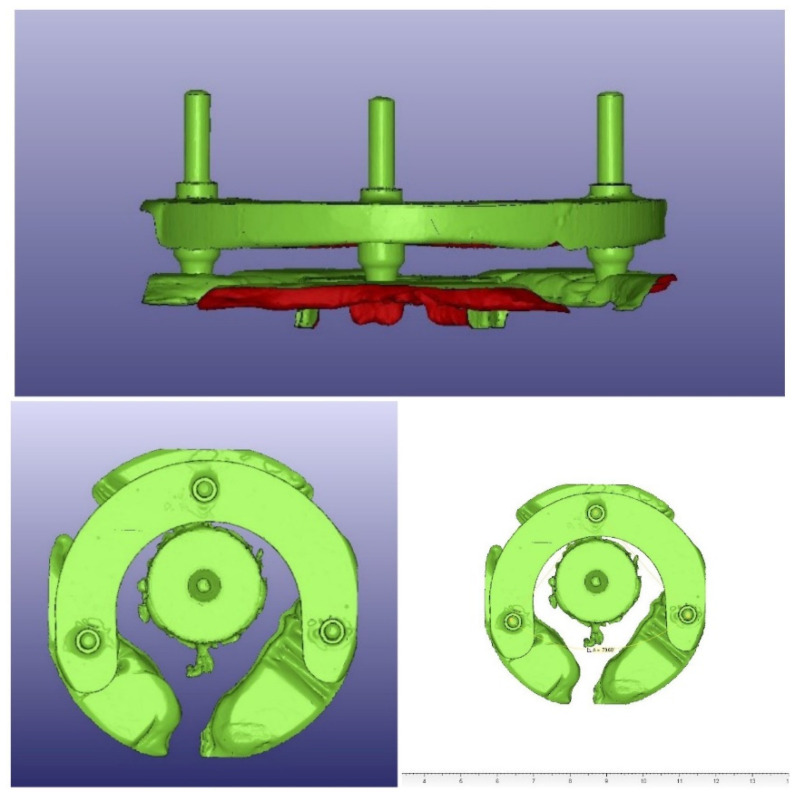
Angulation scans and angulation measurements.

For the measurements of the angulation, three metal rods were connected to three external identical implant replicas and then inserted to the surgical guide. The surgical guides were then scanned (D900 3D scanner; 3Shape), and the resulting STL files were exported to a CAD/CAM analyzing and processing software (VisCAM View V5.2; Marcam Engineering, Bremen, Germany) to digitally measure the three angles between the metal rods for each specimen. Each angle was measured ten times, and then the mean value was calculated for each. Angulation scans and angulation measurements are illustrated in Figure 7.

The surgical guides were first disinfected in disinfection medium for 15 min (Mucalgin; Merz Dental, Lütjenburg, Germany) and then sterilized in a steam sterilizer (6-9-6 HS 2; Belimed, Zug, Switzerland) at 121 °C for 20 min under a pressure of 2 bar [20].

The after-sterilization measurements were conducted 48 h after sterilization for all surgical guides after they were repositioned onto the test model, first with the standardized load of 500 g applied in the same manner as initially. The vertical discrepancies between the guide and the test model were checked again under an optical microscope (Wild M420, 16×) for all 11 marked points for each specimen, and the resulting measurements after sterilization at a load of 500 g (P2) were calculated. Additionally, the specimens were repositioned again, if applicable, with an increased load of up to 1800 g, and the vertical discrepancies between the guides and the test model were checked again for all 11 marked points and the corresponding measurements were calculated (P3).

The metal rods connected to the external identical implant replicas were inserted to the surgical guide. The surgical guides were then scanned again. The resulting STL files were exported to the analyzing software. The three angles between the metal rods were digitally measured after sterilization by means of the same software; each angle was measured ten times, and then the mean was calculated for each.

The collected data were tabulated, coded and introduced to a PC using Statistical Package for Social Science (SPSS 20.0 for windows; SPSS Inc., Chicago, IL, USA, 2011). The data were checked for normal distribution using the Shapiro–Wilk test. The vertical discrepancy values were normally distributed, whereas the variations in discrepancies, the angle values and the variations in the angle measurements were not normally distributed. Mauchly’s sphericity test and Friedman’s test were conducted for the analysis of space values regarding the different measurements for each material as dependent related samples. For the analysis of the material’s influence on the resulting vertical discrepancy, a one-way ANOVA test was conducted followed by the Games–Howell test for pairwise comparisons. For the analysis of statistical differences in the discrepancy variations before and after sterilization with the used material as a variable, the Kruskal–Wallis test was conducted followed by the Mann–Whitney test. For the analysis of statistical differences between the measured angles before and after sterilization, Wilcoxon’s test was performed. For the analysis of statistical differences in the angle variations before and after sterilization with the used material as a variable, the Kruskal–Wallis test was conducted.

## 3. Results

Regarding the initial fit accuracy before the sterilization process, group C showed a significantly smaller vertical discrepancy between the guides and the test model with a mean value of 58 ± 11 µm. Groups S, N and V followed and were statistically comparable. Group L showed significantly the largest vertical discrepancy with a mean value of 254 ± 5 µm.

All the specimens were repositionable after sterilization. The P2 mean values ranged from a minimum of 623 ± 229 µm for group V to a maximum of 1910 ± 552 µm for group L. The P3 mean values ranged from a minimum of 273 ± 74 µm for group V to a maximum of 509 ± 131 µm for group L. The P2 values were statistically higher than P1 values. However, with an applied load up to 1800 g, the P3 values were statistically comparable to the P1 values, although they were still higher than P1 values. Group L had statistically the highest discrepancy after sterilization for the P2 and P3 measurements. Table 2 shows the mean and standard variation values of the measured vertical discrepancies for each group at the three defined measuring conditions.

**Figure 8:**
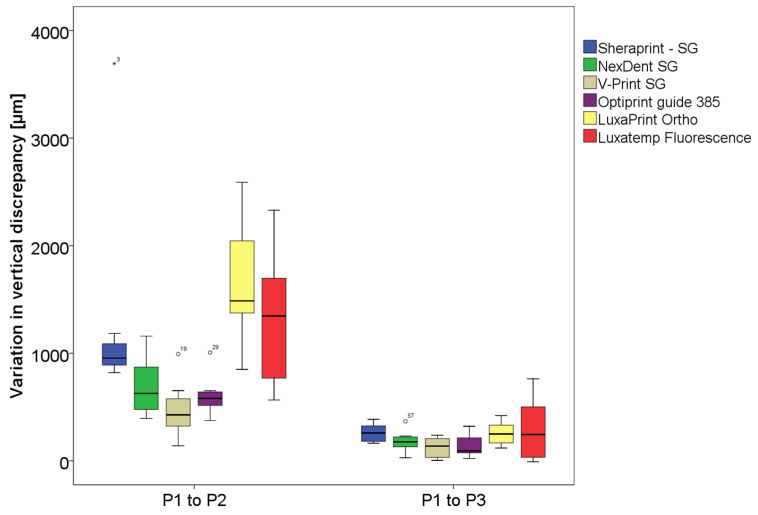
Diagram of the variation in measured vertical discrepancy before sterilization positioned with 500 g (P1) and after sterilization with 500 g (P2) and 1800 g (P3).

The smallest variation in discrepancy at a 500 g load was measured for group V with a median value of 428 µm, whereas the highest was measured for group L with 1487 µm. With applying an increased force to a maximum of 1800 g, the smallest measured variation was for group O with a median value of 94 µm, while the highest was for group S with 260 µm. The choice of material did not influence the variation in the discrepancy when applying an increased load when repositioning the surgical guides on the model after sterilization. The median values of the variation in discrepancy for each group between the initial measured space and the spaces measured after sterilization in µm are shown in Table 3 and illustrated in Figure 8.

There was no statistical difference between the measured angles before and after sterilization for the same material, and therefore no influence of the tested materials on the measured angles could be verified. The variation in the three measured angles was not significant before and after sterilization with a maximum median variation of 1.6°. The median values for the measured angles and the angle variation are shown in Table 4 and Table 5, respectively.

## 4. Discussion

Guided implantology is becoming a commonplace procedure. This is especially beneficial since practicing implant dentistry is no longer limited to highly trained dentists or dental surgeons. Instead, dentists with varying levels of expertise and professional skills can place implants successfully [1]. Surgical guides used for implantation procedures come into contact with the open surgical wound area, bone and blood stream. Performing a high level of disinfection can eradicate many pathogens and microorganisms but not highly potent levels of bacterial spores [30,31]. This necessitates that surgical guides should be categorized as class IIb medicinal products that must be sterile for the surgical procedure, like all other instruments used in implant surgery [32]. 

Nevertheless, some of the most-used 3D-printing materials available on the dental market are classified as class I medicinal products [33,34], while some are categorized as class IIa medicinal products [35]. Regarding the infection control recommendations of manufacturers, the recommendations vary from the disinfection to the sterilization of 3D-printed surgical guides with no defined procedure [33,34,35,36,37].

In this study, five 3D-printing materials for surgical guides were tested. Two of these materials were categorized by the manufacturer as class I medical products; Sheraprint-SG, Shera Werkstoff-Technologie GmbH & Co. KG and NextDent SG, 3D Systems GmbH. One of the tested materials was categorized as a class IIa medicinal product; V-Print SG, Voco. For the other tested materials, no classification was designated by the manufacturer; Optiprint guide 385, Dentona AG and LuxaPrint Ortho, DMG Chemisch-Pharmazeutische Fabrik GmbH. Of the five chosen materials, sterilization was recommended for three materials. For Sheraprint-SG, sterilization was recommended either at 121 °C for 15 min or at 138 °C for 3 min with no mention of the required pressure. For LuxaPrint Ortho, sterilization at 134 °C for 5 min under a pressure of 2 bar was suggested. For V-Print SG, sterilization at 134 °C for a maximum of 5 min under a pressure ranging from 2.07 to 2.17 bar was recommended. For Optiprint guide 385, a disinfection bath was recommended with no mention of sterilization. For NextDent SG, disinfection was also recommended, with sterilization of the material being possible according to the manufacturer with no defined criterion. 

The guides were 3D-printed for each material according to the specified instructions and using the 3D printer recommended by the manufacturer to ensure the optimal production and optimal fit of the surgical guides. Guides made from a Bis-Acrylat self-curing composite (Luxatemp-Flurescence, DMG) were used as the control group. As there was no defined sterilization protocol for all the tested materials, the standard defined protocol applied at the clinic of the authors was followed, and therefore all the guides were first disinfected and then sterilized in an autoclave at 121 °C for 20 min under a pressure of 2 bar. 

The first null hypothesis of this study was partially rejected, as dimensional changes were detected after sterilization for all the tested materials. These changes occurred in the measured vertical discrepancy level and in the measured angulation level. However, the changes in the three measured angulations as well as the angulation variations were not statistically significant (*p* > 0.05). Regarding the measured vertical discrepancy between the surgical guides and the test model, the changes were statistically significant for all the tested materials when comparing the P1 and P2 mean values. However, with the exception of group L, there were no statistical differences when comparing the P1 and P3 mean values after applying a load of 1800 g when repositioning the surgical guides onto the test model. The variation in the discrepancy between P1 and P3 was also statistically not significant (*p* > 0.05). 

This result agrees with the results of a pilot study focusing on the effects of sterilization on the dimensional changes and mechanical properties of 3D-printed surgical guides [32]. In the aforementioned study, one group of surgical drills was sterilized in an autoclave at 121 °C for 20 min under a pressure of 1 bar. Another group was sterilized at 134 °C for 10 min under a pressure of 2 bar. The statistical analysis did not detect any significant deformation after electron-microscopic examination, stereomicroscopic examination and X-ray visibility when comparing the results of both groups with those of a reference group that did not undergo any sterilization. Regarding the mechanical properties, no statistical effects were detected on the flexural strength nor on the compressive strength between the groups. However, for the group that underwent sterilization at 134 °C, the difference in hardness measurements after sterilization was statistically significant [32]. 

The results of the current study were also consistent with the results of another study concentrating on the effect of steam heat sterilization at 121 °C for 20 min on the accuracy of in-office 3D-printed and laboratory 3D-printed surgical guides [38]. This previous study concluded that steam-heat sterilization had no significant effect on the dimensional changes in the tested guides and that no statistical differences were detected between the in-office and laboratory 3D-printed surgical guides [38]. Another study focusing on volumetric changes as well as morphological changes in 3D-printed orthognathic splints as well as surgical cutting guides after sterilization by steam heat or by plasma gas showed that there were no significant differences regarding the volumetric changes for both the tested objects and for both sterilization methods [29]. However, a significant deformation was only detected for the orthognathic splints, and it was significantly distinct after heat sterilization in comparison to the deformation detected after the treatment with gas plasma [29]. This contradiction might be explained by the different 3D-printing methods, i.e., PolyJet 3D-printing in the aforementioned study vs. DLP 3D-printing in the current study, the different tested materials, the different types of specimens and the different assessment methods. A recent study focusing on the accuracy of steam-sterilized printed surgical guides concluded that, at the implant base level, a high and significant deviation in the angle as well as in the 3D accuracy was measured after steam sterilization [39]. This study tested polylactide and polyhydroxyalkanoate surgical guides printed with the fused filament fabrication process and conducted a different methodology [39]. Another study tested the linear dimensional accuracy of SLA surgical guides, in contrast to our study, where all the guides were printed by the DLP process [40]. The study concluded that a significant linear change was detected after sterilization, whereas the group with surgical guides following only a disinfection with 2% glutaraldehyde showed no significant linear dimensional change [40]. 

The second null hypothesis of this study was also partially rejected. The choice of material did not affect the angulation aspect. However, it did affect the measured vertical discrepancy between the guides and the test model. The smallest initial vertical discrepancy measured before the sterilization process was for group C, whereas the greatest was measured for group L. When a standard load of 500 g was applied to reposition the guides after sterilization, the smallest measured space was for group V. Yet, it was statistically comparable with the measured space for all the other groups except for group L, which showed a statistically higher discrepancy. With the application of a load up to 1800 g for the repositioning, the results were similar, with the smallest space measured for group V and the highest measured for group L. Regarding the variation in the discrepancy between P1 and P2, group V showed statistically the smallest variation between P1 and P2, but it was statistically comparable to the variation measured for groups N and O. The variations measured for group L were the highest. Nonetheless, the variations between P1 and P3 were statistically comparable for all the tested materials. 

Group C showed the smallest initial vertical discrepancy and was significantly more accurate in comparison to all five 3D-printing materials. After sterilization, the P2 and P3 values for group C were statistically comparable to the lowest measured vertical discrepancy, respectively. The variation between P1 and P3 for group C was also comparable to those of all the other tested materials. The behavior of the material in accuracy terms could be defined as remarkable, taking into consideration the thermosensitivity of the material [17].

Steam sterilization might not only cause geometric and dimensional changes, as investigated in the current study, but it might also have an effect on mechanical properties. A recent study by Pop et al. investigated the effects of disinfection and steam sterilization (at 121° and at 134°) on the mechanical properties of 3D-printing materials and 3D-printed surgical guides for orthodontic mini-implants [41]. The first part of the study, which investigated the mechanical properties, concluded that DLP 3D-printed specimens that underwent steam sterilization at 121° showed a significant increase in flexural strength, the flexural modulus of elasticity, tensile strength and the tensile modulus of elasticity in comparison to the control group. The second part of the study investigated 3D-printed surgical guides for orthodontic mini-implants and concluded that the DLP 3D-printed guides that underwent steam sterilization at 121° showed a significant increase in the maximum compressive load [41]. Another study reported a 4% decrease in compressive forces and a 7% decrease in the maximum flexural force in the case of bridging element samples for a group of surgical guides that underwent steam sterilization at 121 °C [32].

There are several limitations to this study. The use of a customized simplified test model with no anatomical morphology and with only three implants presents a limitation to the current study as it does not accurately mimic a clinical situation but was designed to facilitate the measurement of this particular laboratory study. The use of the 3D printer recommended for each material by the manufacturer presents another limitation to the current study, as extra variables (printer resolution, printing software, printing parameters, protocol and angulation) might have contributed to additional discrepancies in the measurements. The small sample size for each group could be considered as another limitation to the current study. A distortion analysis could have enriched the results of this study.

## 5. Conclusions

In this laboratory study, the accuracy of 3D-printed surgical guides after steam sterilization at 121 °C in an autoclave was evaluated. A simplified test model was custom-made for this purpose, and the accuracy of these guides was tested for five different 3D-printing materials in addition to a self-curing composite material as a reference in terms of vertical discrepancies and angulation. After sterilization, all the surgical guides were repositionable onto the test model. Significant dimensional changes in terms of increased vertical discrepancy were detected for all the materials after the sterilization process, although these changes were still acceptable when a clinically acceptable increased load was applied while repositioning the guides onto the test model. The choice of material significantly influenced the accuracy of the surgical guides.

## Figures and Tables

**Table 1:** Group codes, compatibility, composition and printer.

Group	Product	Wavelength Compatibility	3D Printer	Composition (in wt. %)
S	Sheraprint-SG	385 nm/405 nm	SHERAprint 30	- Methacrylat oligomere: >90% - Phosphine oxide: <3%
L	LuxaPrint Ortho	385 nm/405 nm	DMG 3Delux	- (Meth) acrylate-based light-curing resin
V	V-Print SG	385 nm	Solflex 350	- BIS-EMA: 50–100% - Urethanedimethacrylate: 10–25% - Diphenyl (2,4,6-trimethylbenzoyl) phosphine oxide: ≤2.5%
O	Optiprint guide 385	385 nm	Asiga Max	- Bisphenol A-ethoxylat (2EO/Phenol) Dimethacrylat
N	NextDent SG	405 nm	NextDent 5100	- Methacrylic oligomers: >90 - Phosphine oxides: <3 - Colorants and pigments
C	Luxatemp-Flurescence	-	-	- Bis-Acrylat self-curing composite

**Table 2:** The mean values and standard deviations of the measured vertical discrepancies for each group at the three defined measuring conditions.

Group	Mean ± SD (µm)		
	Before Sterilization (500 g Load)	After Sterilization (500 g Load)	After Sterilization (1800 g Load)
S	144 ± 68 ^bc, A^	1100 ± 106 ^ab, B^	419 ± 120 ^ab, AB^
N	125 ± 30 ^b, A^	814 ± 275 ^a, B^	308 ± 102 ^a, AB^
V	149 ± 52 ^bc, A^	623 ± 229 ^a, B^	273 ± 74 ^a, AB^
O	216 ± 48 ^cd, A^	823 ± 186 ^a, B^	290 ± 126 ^a, A^
L	254 ± 5 ^d, A^	1910 ± 552 ^b, B^	509 ± 131 ^b, AB^
C	58 ± 11 ^a, A^	1374 ± 610 ^ab, B^	348 ± 284 ^ab, A^

Mean values with the same capital upper-script letters within the same row are not statistically different, *p* > 0.05 (Mauchly’s sphericity test and Friedman’s test). Mean values with the same small upper-script letters within the same column are not statistically different, *p* > 0.05 (one-way ANOVA test followed by Games–Howell’s test). Level of significance α = 0.05 (95% confidence interval).

**Table 3:** Median variation in discrepancy for each group between initial measured space and spaces measured after sterilization in µm.

Group	Median Values (µm)	
	Variations between P1 & P2	Variations between P1 & P3
S	955 _cd_	260 _a_
N	638 _abc_	178 _a_
V	428 _a_	138 _a_
O	581 _ab_	94 _a_
L	1487 _d_	250 _a_
C	1347 _bcd_	245 _a_

Median values with the same small lower-script letters within the same column are not statistically significantly different, *p* > 0.05 (Kruskal–Wallis’s test followed by Mann–Whitney’s test). Level of significance α = 0.05 (95% confidence interval).

**Table 4:** Median angulations for each group between initial situation and after sterilization.

Group	Median Values of Measured Angles (°)					
	Before Sterilization			After Sterilization		
	Angle 1	Angle 2	Angle 3	Angle 1	Angle 2	Angle 3
S	79.7	49.8	50.7	80.1	50.1	50.0
N	79.5	49.8	50.0	80.8	49.9	50.0
V	79.9	50.0	50.5	80.2	49.5	50.3
O	79.7	50.4	50.1	80.8	49.8	49.4
L	80.4	50.1	49.8	79.8	49.5	50.2
C	80.0	50.0	49.6	80.8	49.6	49.8

No statistically significant differences (*p* > 0.05) for measured angle before and after sterilization (Wilcoxon’s test). Level of significance α = 0.05 (95% confidence interval).

**Table 5:** Median values of angulation variation for each group between initial situation and after sterilization.

Group	Median Values of Angle Variation before and after (°)		
	Angle 1	Angle 2	Angle 3
S	0.9	1.6	0.7
N	1.0	1.0	1.2
V	1.1	0.8	1.2
O	1.2	1.0	0.4
L	1.1	1.4	0.9
C	1.4	1.1	1.1

No statistically significant differences (*p* > 0.05) for angulation variations before and after sterilization (Kruskal–Wallis’s test). Level of significance α = 0.05 (95% confidence interval).
